# Lateral transfer of tetrahymanol-synthesizing genes has allowed multiple diverse eukaryote lineages to independently adapt to environments without oxygen

**DOI:** 10.1186/1745-6150-7-5

**Published:** 2012-02-01

**Authors:** Kiyotaka Takishita, Yoshito Chikaraishi, Michelle M Leger, Eunsoo Kim, Akinori Yabuki, Naohiko Ohkouchi, Andrew J Roger

**Affiliations:** 1 Japan Agency for Marine-Earth Science and Technology (JAMSTEC), Yokosuka, Kanagawa, 237-0061, Japan; 2 Centre for Comparative Genomics and Evolutionary Bioinformatics, Department of Biochemistry and Molecular Biology, Dalhousie University, Halifax, Nova Scotia, B3H 1X5, Canada

**Keywords:** eukaryotes, lateral gene transfer, phagocytosis, sterols, tetrahymanol

## Abstract

Sterols are key components of eukaryotic cellular membranes that are synthesized by multi-enzyme pathways that require molecular oxygen. Because prokaryotes fundamentally lack sterols, it is unclear how the vast diversity of bacterivorous eukaryotes that inhabit hypoxic environments obtain, or synthesize, sterols. Here we show that tetrahymanol, a triterpenoid that does not require molecular oxygen for its biosynthesis, likely functions as a surrogate of sterol in eukaryotes inhabiting oxygen-poor environments. Genes encoding the tetrahymanol synthesizing enzyme squalene-tetrahymanol cyclase were found from several phylogenetically diverged eukaryotes that live in oxygen-poor environments and appear to have been laterally transferred among such eukaryotes.

**Reviewers:**

This article was reviewed by Eric Bapteste and Eugene Koonin.

## Findings

A large fraction of eukaryotes and bacteria possess sterols and hopanoids respectively that function as potent stabilizers of cell membranes. Sterols are also associated with fluidity and permeability of eukaryotic cell membranes, and are key to fundamental eukaryotic-specific cellular processes such as phagocytosis [e.g. [1,2]]. Several steps of *de novo *sterol biosynthesis require molecular oxygen [3]. For example, the epoxidation of squalene is the first oxygen-dependent step in the sterol pathway; the epoxidized squalene is then cyclized to either lanosterol or cycloartenol by the enzyme oxidosqualene cyclase (OSC). In contrast, prokaryotic hopanoid biosynthesis does not require molecular oxygen as a substrate, and the squalene is directly cyclized by the enzyme squalene-hopene cyclase (SHC) [4].

Until now, it was unclear how bacterivorous unicellular eukaryotes that are abundant in anoxic or low oxygen environments can carry out phagocytosis. These eukaryotes cannot obtain sterols from food bacteria as the latter generally lack them and sterols cannot be synthesized *de novo *in the absence of molecular oxygen. Explanations that seem plausible are: 1) anaerobic eukaryotes could acquire free sterols from the environment, or 2) they could anaerobically synthesize sterol-like molecules *de novo *using alternative biochemical pathways. Here we provide evidence for the latter by showing that the molecule tetrahymanol is synthesized by anaerobic/microaerophilic eukaryotes and functions as an analogue of sterols in these organisms.

Tetrahymanol is a triterpenoid with five cyclohexyl rings that does not require molecular oxygen for its synthesis. It was first discovered in the ciliated protozoan *Tetrahymena pyriformis *[5] but has been more recently detected in other ciliates, the anaerobic rumen fungus *Piromonas *(*Piromyces*) *communis*, the fern *Oleandra wallichii*, the purple nonsulfur bacterium *Rhodopseudomonas palustris*, and the nitrogen-fixing bacterium *Bradyrhizobium japonicum*, although in *O. wallichii *and *B. japonicum *this compound is only a trace constituent of their lipidome [6-10].

Through surveys of expressed sequence tags (EST) and genome data from a broad range of eukaryotes, the genes similar to the tetrahymanol-synthesizing (squalene-tetrahymanol cyclase: STC) genes of the ciliates *Tetrahymena *and *Paramecium *were identified from *Piromyces *sp., *Sawyeria marylandensis, Andalucia incarcerata, Trimastix pyriformis*, and *Alvinella pompejana*. Because the deduced amino acid sequences of the proteins we have newly identified share a conserved amino acid residue possibly involved in the formation of the backbone structure characteristic to tetrahymanol with those of the ciliate STCs, they all likely possess the same function as the STC enzymes of ciliates (see additional file 1).

*S. marylandensis, A. incarcerata, T. pyriformis *belong to the eukaryotic 'super-group' Excavata and are all anaerobic/microaerophilic free-living protists (single-celled eukaryotes) that possess mitochondrion-related organelles similar to hydrogenosomes [11-13]. These protists feed almost exclusively on bacterial prey. *A. pompejana *is a polychaete worm found only at deep-sea hydrothermal vents where it frequently encounters anoxic/hypoxic conditions due to reducing hydrothermal fluids. These animals harbor bacterial episymbionts on their dorsal surfaces that they likely consume as food [14]. As molecular oxygen is not required for the biosynthesis of tetrahymanol [15], it is reasonable to assume that these eukaryotes inhabiting oxygen-poor environments produce tetrahymanol rather than sterols.

**Figure 1:**
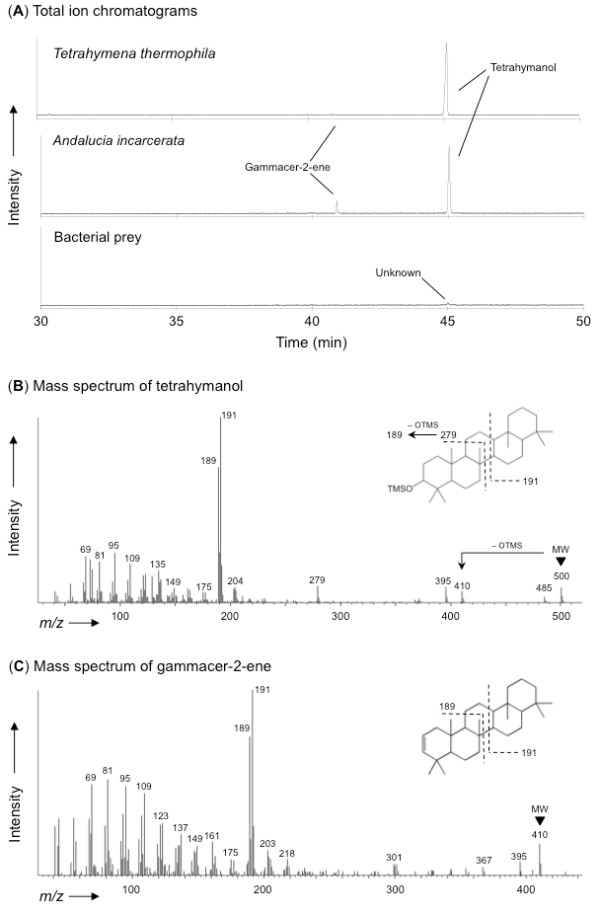
**Gas chromatography/mass spectrometry (GC/MS) of lipids from *Tetrahymena thermophila *and *Andalucia incarcerata***. (A) Total ion chromatograms of lipid extracts as trimethylsilyl (TMS) derivatives obtained by GC/MS analyses, and mass spectrum for (B) tetrahymanol and (C) gammacer-2-ene. Tetrahymanol and gammacer-2-ene were identified by coincidence in the mass spectra of the previous study [21]. Both tetrahymanol and gammacer-2-ene were obtained in the lipid extracts from *Tetrahymena thermophila *and *Andalucia incarcerata*, but not in those from bacterial prey of *A. incarcerata*. Although an unknown peak was observed in the chromatogram of bacterial prey with the retention time (44.7 min), the mass spectrum of this peak was completely different from that of tetrahymanol. No peaks corresponding to those of sterols were found in the chromatograms of *T. thermophila, A. incarcerata*, and bacterial prey of *A. incarcerata*.

To test the hypothesis that these genes we found actually encode STC, we used gas chromatography/mass spectrometry (GC/MS) on a lipid fraction of the anaerobically cultured cells of *A. incarcerata *(experimental procedures are in additional file 2), and we determined that sterols were completely absent and that tetrahymanol was present (Figure 1). Gammacer-2-ene was also detected in *A. incarcerata *as well as *Tetrahymena thermophila *as a minor component, but it is uncertain whether this compound naturally occurred in the cells or was artificially generated from tetrahymanol during the GC/MS experiment. The bacterial prey cells of *A. incarcerata *coexisting in the medium were overwhelmingly dominated by *Vibrio *sp., *Fusibacter *sp., *Bacteroides *sp., and *Arcobacter *sp. (identified by ribosomal RNA typing: see additional file 3) and were confirmed to lack tetrahymanol by GC/MS (Figure 1).

Ciliates such as *Tetrahymena *and *Paramecium *are aerobes, and so it remains unclear why they would synthesize tetrahymanol rather than sterol (there are no genes for OSC and the enzyme associated with squalene epoxidation at least in the genomes of these two ciliates). There may be species with a cryptic life-cycle stage occurring in an oxygen-poor environment among ciliates known to be aerobes [16] or it may be that they transiently encounter low-oxygen pockets in their environments (or their common ancestor did). Either situation would provide sufficient selective benefit favouring the production of tetrahymanol over sterols.

**Figure 2:**
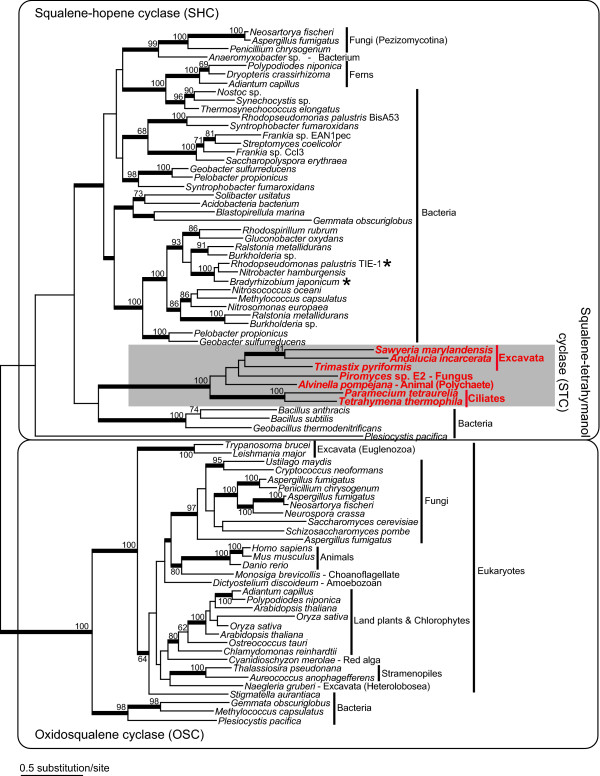
**A maximum-likelihood phylogeny based on the sequences of oxidosqualene cyclase (OSC), squalene-hopene cyclase (SHC), and squalene-tetrahymanol cyclase (STC) from a broad range of organisms**. The STC clade is shaded. The tetrahymanol-synthesizing bacteria *Rhodopseudomonas palustris *strain TIE-1 and *Bradyrhizobium japonicum *are marked with an asterisk (there is no data whether *R. palustris *strain BisA53 produces tetrahymanol or not). Bootstrap probabilities are shown for nodes with support over 50%. Thick branches represent nodes supported by Bayesian posterior probabilities over 0.95. Ferns and pezizomycete fungi likely acquired the SHC genes from a cyanobacterium and *Anaeromyxobacter *via lateral gene transfer, respectively, while the OSC genes of the few bacteria are of possible eukaryotic origin.

To probe the origins of the STC enzymes in anaerobic eukaryotes, we conducted phylogenetic analyses (methods are described in additional file 4). The maximum-likelihood (ML) phylogenetic tree shown in Figure 2 depicts two major (OSC and SHC) clades separated by a long internal branch. The STC sequences from phylogenetically diverged eukaryotes (ciliates, excavates, a fungus, and an animal) formed a monophyletic group within the SHC radiation with 100% ML bootstrap support and a posterior probability of 1.00 in Bayesian analysis. The monophyly of eukaryote STC homologues could be explained by vertical inheritance from a common tetrahymanol-synthesizing eukaryotic ancestor. However, if this were the case, dozens of parallel losses of the STC genes in many eukaryotic lineages that currently lack this gene would be required, so this evolutionary scenario seems unlikely. An alternative and more plausible possibility is that the STC gene has been laterally transferred among phylogenetically diverged eukaryotes through an unknown mechanism, providing a selective benefit to those organisms adapting to anoxic/hypoxic environments. However, the putative original donor of the STC gene could not be confidently identified in this study because most branches within the STC clade were not resolved well and the STC clade did not strongly affiliate with any OSC and SHC sequences including those of tetrahymanol-synthesizing bacteria *Rhodopseudomonas *and *Bradyrhizobium *(although the STC clade showed some weak affinity to the *Bacillus*/*Geobacillus *group with 48% ML bootstrap support and 0.85 posterior probability in Bayesian analysis). This is consistent with previous phylogenetic analyses based on all sequences of the triterpene cyclase protein family (including OSC, SHC, and STC) available from public databases that failed to identify sequences closely related to the sole eukaryotic (ciliate) STC known at that time [17].

Most bacteria are not capable of phagocytosis or similar endocytic processes. However, the planctomycete *Gemmata obscuriglobus*, which exceptionally synthesizes sterols, has an endocytosis-like protein uptake process [18], further supporting the hypothesis that sterols (or sterol-like molecules) are required for phagocytosis/endocytosis. That sterol-lacking, tetrahymanol-synthesizing eukaryotes (at least ciliates and *A. incarcerata*) are phagocytic strongly suggests that tetrahymanol is functionally replacing sterols in facilitating this process. In fact, it has been demonstrated that sterol supplementation results in the repression of tetrahymanol production in *Tetrahymena *[19]. These findings also suggest that the original bacterial donors of the STC gene, and perhaps their living descendants, if found, may also utilize phagocytosis-like processes as in the case of *G. obscuriglobus*.

Our findings may have important implications for the field of geochemistry. Eukaryotes first arose in the Proterozoic (i.e. from 2.5 to 0.54 Ga ago) at a time when the oceans were likely oxygenated in shallow-water settings and were anoxic and sulfidic in deep-water [20]. At that time, eukaryotes adapting to these vast oxygen-poor environments may have also acquired the STC gene and synthesized tetrahymanol; it is quite possible that such eukaryotes were more abundant both in terms of diversity and biomass than extant tetrahymanol-using taxa. Tetrahymanol is the precursor of the molecular fossil biomarker gammacerane [21] that has frequently been associated with oxygen-poor environments such as stratified water columns and has, so far, been detected in formations as old as the 2.67-2.46 Ga Transvaal Supergroup sediments [22]. The biological origins of gammacerane are still incompletely understood. Our results suggest that gammacerane may be at least partially derived from eukaryotes that may have inhabited the vast low-oxygen marine environments of the Proterozoic.

## Competing interests

The authors declare that they have no competing interests.

## Authors' contributions

KT and AJR conceived and designed the experiments: KT, YC, MML, EK and AY performed the experiments: KT and YC analyzed the data: KT, NO and AJR contributed reagents/materials/analysis tools. KT, AJR, YC and NO wrote the paper. All authors read and approved the final manuscript.

## Reviewers' comments

### Reviewer 1: Dr. Eric Bapteste

In my view this article can be accepted for publication in Biology Direct. I would nonetheless appreciate if the authors would attempt some further investigations regarding the origin(s) of the transferred STC genes.

In particular, I would be curious to know more about the possible mechanisms involved in these transfers. How was the first STC gene acquired by eukaryotes if this gene is required for phagocytosis? It could not be through predation then... Were the first eukaryotes who acquired this gene non phagocytic ones, or did this gene come to replace another (non orthologous) gene fulfilling this function? For the more recent STC gene acquisitions: is there any evidence in the sequences from known (eukaryotic) viruses that the STC gene may have been mobilized by viruses? Would it be possible to check in (some of) the eukaryotic genomes owning the STC gene whether some traces of transposon activity can be found next to the acquired STC gene? Is there a distant homologue of the STC gene in the mitochondria: could this gene have been transferred internally from that symbiont, or using that symbiont as an 'entry door' into the eukaryotic cell?

*Authors' response*: Considering the fact that the sterol biosynthesis pathway is broadly distributed across phylogenetically divergent eukaryotes, it is very likely that the common ancestor of extant eukaryotes already possessed this metabolic pathway (see references [3] and [23]). Thus, it is reasonable to assume, as we do, that the first (putative aerobic or facultatively anaerobic) eukaryote that acquired the STC gene from a bacterium was a normal sterol-producing phagocytic eukaryote. The acquisition of the STC gene is thought to be one of strategies for secondary adaptation to living exclusively in oxygen-poor environments. Similarly, the hosts for the subsequent eukaryote-to-eukaryote transfers of the STC genes we propose would also originally be able to synthesize sterols and perform phagocytosis/endocytosis. But once they acquired the STC gene, they could easily adapt to living permanently in anoxic/hypoxic environments. The detailed mechanisms mediating the lateral gene transfers (LGTs) that we describe, as with many proposed LGTs in eukaryotic genomes, remain unclear. We could not find any traces of putative LGT vectors such as transposable elements in the flanking regions of the STC genes in the genomes from the ciliates *Tetrahymena *and *Paramecium*. Furthermore, since the other STC genes found in this study were retrieved from EST data, their non-transcribed genomic flanking regions remain uncharacterized at this time. Finally, as far as we know, there is no distantly-related STC homologue encoded in any mitochondrial genome characterized to date. Thus, there is no evidence linking our findings to endosymbiotic gene transfer from the mitochondrial (or plastid) ancestor and no other reason to think these organelles are relevant to this case of gene transfer.

I also wonder what is the general 'availability' of the STC genes in the environment. Are there STC genes everywhere (which would enhance the chance of some needy eukaryotes to pick them anywhere), or is their distribution somehow restricted to some environments (in which we could then imagine that the transfer had more chances to occur)? It would be interesting to see what the general distribution of this gene is based on sequences in publicly available metagenomic datasets.

*Authors' response*: This is an interesting point. We did investigate this, however, the STC gene could not be found in publicly available metagenomic datasets including anoxic/hypoxic ones. Such absence of this 'eukaryotic' gene is probably due to the overwhelming dominance of prokaryotic sequences in the metagenomic datasets. It is currently impossible to rigorously address the issue the reviewer has raised. However, metagenomic sequencing efforts that are targeted at eukaryotic microbes specifically may allow us to address this in future.

Finally, the very last section of the paper could be slightly toned-down. I would prefer that the authors suggest that their 'findings MAY (my suggestion) have important implications for the field of geochemistry' rather than their 'findings have important implications for the field of geochemistry'.

*Authors' response*: Indeed, we were perhaps too enthusiastic in this section. Accordingly we have taken the reviewer's advice.

### Reviewer 2: Dr. Eugene V. Koonin

This is an excellent study that solves a real biological puzzle: how anaerobic eukaryotes do without sterols and in particular how do they manage to perform phagocytosis? It is particularly impressive that, after coming up with the hypothesis that tetrahymanol is the functional analog of sterols in these organisms, on the basis of genome and EST sequence analysis, the authors actually discover this molecule directly. The phylogenetic analysis presented in the paper is compelling. It is an important addition to the existing understanding of the evolution of eukaryotes. There is nothing to criticize.

*Authors' response*: We thank the reviewer for his kind comments.

## Supplementary Material

Additional file 1 **Partial amino acid alignment of squalene-tetrahymanol cyclase, squalene-hopene cyclase, and oxidosqualene cyclase**. The amino acid residues at the 608 position of squalene-tetrahymanol cyclases associated with the C-ring carbocation are histidine, while those of most squalene-hopene cyclase and oxidosqualene cyclase are phenylalanine. Click here for file

Additional file 2 **Gas chromatography mass spectrometry**. *Andalucia incarcerata *as well as *Tetrahymena thermophila *possesses tetrahymanol rather than sterols based on gas chromatography mass spectrometry. Click here for file

Additional file 3 **Identification of bacterial prey in the culture of *Andalucia incarcerata***. The genera *Vibrio, Fusibacter, Bacteroides*, and *Arcobacter *were identified in the culture of *A. incarcerata *by ribosomal RNA typing. Click here for file

Additional file 4 **Genetic analyses of squalene-tetrahymanol cyclase genes**. Procedures of isolation, identification and phylogenetic analyses of squalene-tetrahymanol cyclase genes are described. Click here for file
