# Supplementary material for: Lateral transfer of tetrahymanol-synthesizing genes has allowed multiple diverse eukaryote lineages to independently adapt to environments without oxygen
Source: Biol Direct. 2012 Feb 1;7:5. doi: 10.1186/1745-6150-7-5 (PMC3317845; doi:10.1186/1745-6150-7-5)
Supplement: Additional file 1 — Partial amino acid alignment of squalene-tetrahymanol cyclase, squalene-hopene cyclase, and oxidosqualene cyclase. The amino acid residues at the 608 position of squalene-tetrahymanol cyclases associated with the C-ring carbocation are histidine, while those of most squalene-hopene cyclase and oxidosqualene cyclase are phenylalanine. [file 1745-6150-7-5-S1.PDF]

608

Group	Organism	Sequence
STC	Tetrahymena	GTG**H**RGLLY
	Paramesium	GTG**H**RGLLY
	Piromyces	GTG**H**RGFLM
	Trimastix	GTG**H**PGIIY
	Sawyeria	GTG**H**RKLLY
	Andalucia	GTG**H**RGVLY
	Alvinella	GTG**H**RGVIF
SHC	Gemmata	CTA**F**ASGVE
	Rhodopseudomonas	GTG**F**PGSFM
	Syntrophobacter	GTG**F**PGAFM
	Saccharopolyspora	GTG**F**PGDFY
	Streptomyces	GTG**F**PWDFS
	Frankia	GTG**F**PGDFY
	Pelobacter	GTG**F**PKFFM
	Geobacter	GTG**F**PKFFM
	Syntrophobacter	GTG**F**PKHFY
	Nostoc	GTG**F**PCHFY
	Thermosynechococcus	GTG**F**PCHFY
	Synechocystis	GTG**F**PCHFY
	Methylococcus	APG**F**PRVFY
	Nitrosococcus	APG**F**PRVFY
	Nitrosomonas	APG**F**PKVFY
	Ralstonia	APG**F**PRIFH
	Burkholderia	APG**F**PRIFY
	Nitrobacter	ATG**F**PRVFY
	Rhodospirillum	AVG**F**PRIFY
	Gluconobacter	AVG**F**PKVFY
	Blastopirellula	GTG**F**PRVFY
	Acidobacteria	GTG**F**PRVFY
	Solibacter	GTG**F**PRVFY
	Anaeromyxobacter	GTG**F**PRHFY
	Bradyrhizobium	ATG**F**PRVFY
	Bacillus	GIG**L**PKQFY
	Geobacillus	GQG**M**AGAFY
	Plesiocystis	AP-**L**PGYGL
OSC	Trypanosoma	GV-**F**NGNNP
	Cryptococcus	GI-**F**NKNCA
	Aspergillus	GI-**F**NKSCA
	Saccharomyces	GV-**F**NHSCA
	Ustilago	GI-**F**NRNCA
	Cyanidioschyzon	GV-**F**NRNCM
	Aureococcus	GV-**F**NRSCG
	Thalassiosira	GV-**F**NRACG
	Dictyostelium	GV-**F**NFNCM
	Mus	GV-**F**NKSCA
	Homo	GV-**F**NKSCA
	Danio	GV-**F**NKSCA
	Monosiga	GV-**F**NKNCM
	Naegleria	GV-**F**NHNCM
	Ostreococcus	GV-**F**NANCM
	Chlamydomonas	GV-**F**NRNCM
	Arabidopsis	GV-**F**NRNCM
	Oryza	GV-**F**NKNCM
	Stigmatella	GI-**F**NRTCA
	Plesiocystis	GV-**F**FNTAV
	Methylococcus	GV-**F**FGAAM
	Gemmata	GV-**F**FGTAM

694

Figure S1. Partial amino acid alignment of squalene-tetrahymanol cyclase (STC), squalene-hopene cyclase (SHC), and oxidosqualene cyclase (OSC). Gaps are represented by dashes. Numbers above and below the alignment are the amino acid positions in *Nostoc* SHC and *Monosiga* OSC, respectively. The position with a color letter (608/694) is associated with the C-ring carbocation. Sterols, hopanoids, and tetrahymanol all contain a cyclohexyl C ring, which is formed by propagation of the carbocation to the anti-Markovnikov position at C_{13}. Stabilization of the anti-Markovnikov cation at position at C_{13} is strongly dependent on the conserved residue phenylalanine (F) at 608/694 positions of SHC/OSC. Indeed, all known SHCs and OSCs other than those of *Bacillus*, *Geobacillus* and *Plesiocystis* contain the Phe608/694 residue. SHCs from *Rhodopseudomonas palustris* and *Bradyrhizobium japonicum* producing tetrahymanol as well as hopanoids also contain phenylalanine at the corresponding position, although it remains unclear how these bacteria synthesize tetrahymanol. On the other hand, STCs from ciliates exceptionally contains histidine (H) at the corresponding position. This conserved amino acid substitution leads to the provocative suggestion that there may be a different mechanism for ring closure reactions that need to form three anti-Markovnikov cations unique to tetrahymanol (rings C, D, and E). His608 was strictly conserved among the deduced amino acid sequences of the genes similar to STC genes of ciliates found in this study.
