# Supplementary material for: Lateral transfer of tetrahymanol-synthesizing genes has allowed multiple diverse eukaryote lineages to independently adapt to environments without oxygen
Source: Biol Direct. 2012 Feb 1;7:5. doi: 10.1186/1745-6150-7-5 (PMC3317845; doi:10.1186/1745-6150-7-5)
Supplement: Additional file 2 — Gas chromatography mass spectrometry. Andalucia incarcerata as well as Tetrahymena thermophila possesses tetrahymanol rather than sterols based on gas chromatography mass spectrometry. [file 1745-6150-7-5-S2.PDF]

Gas chromatography mass spectrometry (GC/MS)

To have a reference organism to test that contains tetrahymanol, an axenic culture of *Tetrahymena thermophila* was used. *T. thermophila* was grown in PPYG medium (0.4% proteose peptone, 0.2% yeast extracts, and 1% glucose) at 25°C. After harvesting cells of *T. thermophila*, *Andalucia incarcerata* (with bacterial prey), and the bacterial prey of *A. incarcerata* by centrifugation at 3,500 xg for 10 minutes at 4°C, cells were washed twice with phosphate buffered saline. These cells were saponified with 0.5M potassium hydroxide in methanol/water (95/5, w/w) at 75ºC for overnight. After addition of water, neutral lipids (including tetrahymanol) were extracted with *n*-hexane/dichloromethane (2/1, v/v). The lipid extracts were trimethyl silylated using N,O-bis-trimethylsilyl-acetamide at 75ºC for 30 min.

GC/MS analysis was performed using an Agilent Technologies 6890N GC/5973A MSD system with a Gerstel programmable temperature vaporizing (PTV) injector. The lipid extracts as trimethylsilyl (TMS) derivatives were injected into the PTV injector using a solvent vent mode and analyzed on an HP-5MS capillary column (60m length × 0.25 mm i.d., 0.25 mm film thickness). The PTV temperature was programmed as follows: heating from 60°C to 350°C at a rate of 600°C min^{-1} after 0.3 min at the initial temperature, holding isothermally at 350°C for 10 min. The GC oven temperature was programmed as follows: heating from 50°C to 120°C at a rate of 30°C min^{-1} after 2 min at the initial temperature, followed by heating to 310°C at a rate of 6°C min^{-1} and holding isothermally at 310°C for 20 min. Carrier gas (He) flow rate through the GC capillary column was controlled using a constant flow mode at 1.5 mL min^{-1}.
