# Supplementary material for: Lateral transfer of tetrahymanol-synthesizing genes has allowed multiple diverse eukaryote lineages to independently adapt to environments without oxygen
Source: Biol Direct. 2012 Feb 1;7:5. doi: 10.1186/1745-6150-7-5 (PMC3317845; doi:10.1186/1745-6150-7-5)
Supplement: Additional file 3 — Identification of bacterial prey in the culture of Andalucia incarcerata. The genera Vibrio, Fusibacter, Bacteroides, and Arcobacter were identified in the culture of A. incarcerata by ribosomal RNA typing. [file 1745-6150-7-5-S3.PDF]

Identification of bacterial prey in the culture of *Andalucia incarcerata*

To identify the bacterial prey that were co-cultured with *A. incarcerata,* genomic DNA from the harvested cells was extracted with the UltraClean Soil DNA isolation Kit (MO BIO Laboratories) and bacterial 16S rRNA genes were PCR-amplified with the primers (forward: 5'-AGAGTTTGATCCTGGCTCAG-3' and reverse: 5'-GGTTACCTTGTTACGACTT-3'). After the PCR-amplified products were cloned as described above, the sequences of 48 clones were obtained and subsequently analyzed using Blast against the non-redundant database for identification. For gas chromatography mass spectrometry the bacterial prey cells were isolated from the culture of *A. incarcerata* with a capillary pipette and a bacterial culture without *A. incarcerata* was established using the same medium. The bacterial composition of the *A. incarcerata*-free culture was confirmed to be identical to that of the original culture with *A. incarcerata* by clone sequencing. There has been no evidence that any of the bacterial species within the genera *Vibrio*, *Fusibacter*, *Bacteroides*, and *Arcobacter* identified in the culture of *A. incarcerata* by ribosomal RNA typing produce tetrahymanol.

Table S1. Bacterial prey in the culture of *Andalucia incarcerata* identified by ribosomal RNA typing.

Bacterial prey identified	Homology	Clone number
Vibrio sp.	*Vibrio* sp. strain MM03 (99%)	34
Bacteroidetes sp.	*Bacteroidetes* sp. strain Ko710 (98%)	6
Fusibacter sp.	*Fusibacter paucivorans* (95%)	5
Arcobacter sp.	*Arcobacter nitrofigilis* (99%)	3
