# Supplementary material for: Lateral transfer of tetrahymanol-synthesizing genes has allowed multiple diverse eukaryote lineages to independently adapt to environments without oxygen
Source: Biol Direct. 2012 Feb 1;7:5. doi: 10.1186/1745-6150-7-5 (PMC3317845; doi:10.1186/1745-6150-7-5)
Supplement: Additional file 4 — Genetic analyses of squalene-tetrahymanol cyclase genes. Procedures of isolation, identification and phylogenetic analyses of squalene-tetrahymanol cyclase genes are described. [file 1745-6150-7-5-S4.PDF]

Genetic analyses of squalene-tetrahymanol cyclase (STC) genes

We used tBlastX [1] to screen for STC genes in public or the authors' EST data sets and in public genomic data by using the STC sequence of *Tetrahymena thermophila* (GenBank accession number XM_001026696) as a query. Overlapping sequences corresponding to STC transcripts from the same EST data set were assembled into clusters with Sequencher (Gene Codes Corporation).

Because the STC gene fragments from *Trimastix pyriformis* and *Andalucia incarcerata* identified with tBlastX were truncated, their full-length coding sequences were obtained using 5' and 3' rapid amplification of cDNA ends (RACE) methods. Cultivation and RNA extraction of *T. pyriformis* were performed as described in [2]. Cells of *A. incarcerata* were cultivated in 45 ml of sterile seawater mixed 1:1 with Neff's (=Page's) Amoeba Saline, plus 1:30 v/v LB media at 20°C in 50 ml polypropylene tubes, and were harvested by centrifugation at 3,220 g for 10 minutes at 4°C. Total RNA of *A. incarcerata* was isolated using a TRIzol® Reagent-based protocol (Invitrogen). The RACE experiments were performed using exact-match primers based on the identified sequences using the GeneRacer Kit (Invitrogen), for *T. pyriformis,* and the 5'/3'-RACE Kit, 2nd Generation (Roche Applied Science), for *A. incarcerata,* in both cases following the manufacturers' instructions. The PCR-amplified DNA fragments from *T. pyriformis* and *A. incarcerata* were cloned into the pCR21-TOPO (Invitrogen) and pGEM-T Easy (Promega) vectors, respectively, followed by sequencing. The full-length sequences of the STC genes from *T. pyriformis* and *A. incarcerata* have been deposited in GenBank with the accession numbers

AB669021 and AB669022, respectively.

The deduced amino acid sequences of the STC genes identified in this study were aligned with the sequences of OSC and SHC from various eukaryotic and prokaryotic species using ClustalX version 2.0 [3]. The alignments were inspected by eye and manually edited. After exclusion of ambiguously aligned sites, the final dataset included 81 taxa with 442 positions (available upon request from the corresponding author). For this dataset maximum-likelihood (ML) analyses were carried out using PHYML ver. 3.0 [4]. Both SPR and NNI tree-searching algorithms were used with five random starting trees. The amino acid substitutions in the data were modeled under the LG model [5] incorporating the amino acid frequencies estimated from the dataset, a proportion of invariable sites, and among-site rate variation approximated by a discrete gamma distribution with four categories (LG + I + Γ + F model), which was selected as the most appropriate model by the program ProtTest [6] under the Akaike information criterion. We also conducted an ML bootstrap analysis (1,000 replicates) with the same PHYML model settings. Bayesian analysis was also conducted using PhyloBayes ver.3.3 [7] with the LG + Γ + F model. For the PhyloBayes analysis, we used this model, which was selected as a second-best substitution model by ProtTest, because the proportion of invariable sites cannot be considered in this program. Two parallel Markov Chain Monte Carlo runs were run for 20,000 generations, sampling log-likelihoods (lnLs) and trees at 10-generation intervals. The first 4,000 generations were discarded as burn-in and trees were summarized to obtain Bayesian posterior probabilities.

References

1. Altschul SF, Madden TL, Schaffer AA, Zhang J, Zhang Z, Miller W, Lipman DJ: **Gapped BLAST and PSI-BLAST: a new generation of protein database search programs.** *Nucleic Acids Res* 1997, **25:**3389-3402.
2. Hampl V, Silberman JD, Stechmann A, Diaz-Triviño S, Johnson PJ, Roger AJ: **Genetic evidence for a mitochondriate ancestry in the 'amitochondriate' flagellate *Trimastix pyriformis*.** *PLoS One* 2008, **3:**e1383.
3. Thompson JD, Gibson TJ, Plewniak F, Jeanmougin F, Higgins DG: **The CLUSTAL_X windows interface: flexible strategies for multiple sequence alignment aided by quality analysis tools.** *Nucleic Acids Res* 1997, **25:**4876-4882.
4. Guindon S, Dufayard JF, Lefort V, Anisimova M, Hordijk W, Gascuel O: **New algorithms and methods to estimate maximum-likelihood phylogenies: assessing the performance of PhyML 3.0.** *Syst Biol* 2010, **59:**307-321.
5. Le SQ, Gascuel O: **An improved general amino acid replacement matrix.** *Mol Biol Evol* 2008, **25:**1307-1320.
6. Abascal F, Zardoya R, Posada D: **ProtTest: selection of best-fit models of protein evolution.** *Bioinformatics* 2005, **21:**2104-2105.
7. Lartillot N, Lepage T, Blanquart S: **PhyloBayes 3: a Bayesian software package for phylogenetic reconstruction and molecular dating.** *Bioinformatics* 2009, **25:**2286-2288.
